# The synthesis of niclosamide derivative as a novel colistin adjuvant for reversing colistin resistance and its antibacterial activity with colistin *in vitro*

**DOI:** 10.1128/msphere.00593-26

**Published:** 2026-10-08

**Authors:** Peiyi Liu, Yingbiao Li, Fanlun Ding, Dandan He, Yajun Zhai, Li Yuan, Xianya Wang, Guoyu Yang, Gongzheng Hu

**Affiliations:** 1 College of Veterinary Medicine, Henan Agricultural University 70573, Zhengzhou, Henan, China; 2 Xinxiang Hexie Animal Pharmaceutical Co. Ltd., Xinxiang, Henan, China; 3 College of Chemistry and Materials, Henan Agricultural University 70573, Zhengzhou, Henan, China; University of Nebraska Medical Center College of Medicine, Omaha, Nebraska, USA

**Keywords:** colistin, niclosamide derivative (Axilisuan), O-carboxymethylation, multidrug-resistant bacteria

## Abstract

Colistin (COL) serves as a last-line therapeutic agent against multidrug-resistant gram-negative bacteria (GNB) infections. Nevertheless, widespread clinical use of COL has led to severe bacterial resistance. The anthelmintic drug niclosamide (NIC) has been shown to enhance the activity of COL in combination therapy. Despite this synergistic advantage, the two compounds possess distinct physicochemical and pharmacokinetic properties. To enable simultaneous delivery of both drugs to the target site and maximize their synergistic antibacterial activity, we propose the synthesis of a prodrug conjugate linking COL and NIC. As a preparatory step toward a future COL-NIC conjugate, we synthesized and characterized the carboxymethylated derivative of NIC (Axilisuan [AXLS]) to reduce cytotoxicity, improve aqueous solubility, and maintain the NIC synergistic antibacterial effect with COL. AXLS was successfully synthesized and characterized by high-resolution mass spectrometry and proton nuclear magnetic resonance spectroscopy. Solubility studies showed that AXLS exhibited approximately 57-fold higher aqueous solubility than NIC. Both checkerboard assays and time-kill curve analyses indicated that AXLS displayed COL-enhancing activity comparable to that of NIC. Importantly, AXLS showed lower cytotoxicity than NIC, both alone and in combination with COL. Collectively, these findings provide crucial insights for the future development of synthetic strategies toward novel COL-NIC conjugates and offer a rational design framework for the chemical modification of salicylanilide-based antibacterial adjuvants against colistin-resistant GNB.

**IMPORTANCE:**

Colistin remains one of the few treatment options for infections caused by multidrug-resistant gram-negative bacteria, but increasing colistin resistance limits its clinical use. Niclosamide can potentiate colistin activity, but its poor aqueous solubility and safety liabilities limit further development. Here, we synthesized a carboxymethylated niclosamide derivative (Axilisuan) and found that it retained colistin-potentiating activity while showing markedly improved aqueous solubility and reduced *in vitro* toxicity compared with niclosamide. These findings support phenolic hydroxyl modification as a practical strategy for improving salicylanilide-based antibacterial adjuvants and provide a tractable intermediate for future development of colistin-niclosamide conjugates.

## INTRODUCTION

Antimicrobial resistance (AMR), which poses a serious threat to global health, has been declared by the World Health Organization as one of the major global health crises of the 21st century (1–3). In 2019 alone, antibiotic resistance was associated with nearly 5 million deaths worldwide. Without effective intervention, AMR could kill 10 million people per year by 2050. Colistin (COL) is often employed as a last-line agent for infections caused by multidrug-resistant (MDR) organisms. Nevertheless, the rapid dissemination of COL-resistant (COL-R) strains has drastically diminished the utility of this vital antibiotic. With the pipeline of novel antibiotics failing to keep pace with the evolution of resistance, there is an urgent need for adjunctive strategies that either restore the efficacy of existing antibiotics or suppress the development of resistance. Although combination therapy, particularly the co-administration of COL with adjuvant agents, has emerged as a promising strategy to reverse resistance and enhance antimicrobial efficacy, few colistin-potentiating adjuvants have advanced to clinical use due to concerns regarding safety, specificity, efficacy, and dosage requirements (4, 5).

Niclosamide (NIC), a salicylanilide derivative, has been extensively utilized as an antiparasitic agent since the 1960s. It has been shown to possess broad-spectrum biological activities, including antitumor and antiviral effects, while its antibacterial and antifungal properties have attracted increasing attention in recent years (6). Particularly noteworthy is its synergistic efficacy in combination therapies. Multiple *in vitro* studies have demonstrated that the combination of NIC and COL exhibited synergistic antibacterial activity against multidrug-resistant gram-negative bacteria (GNB) (such as *Pseudomonas aeruginosa* and *Acinetobacter baumannii*), effectively reversing bacterial resistance to COL (7, 8). However, the clinical application of this combination is limited due to several pharmacokinetic drawbacks: COL has poor oral absorption, susceptibility to degradation in the stomach, and significant nephrotoxicity and neurotoxicity when administered via injection; meanwhile, NIC is characterized by poor aqueous solubility, low oral bioavailability (approximately 10%), and a short half-life (approximately 45 min) (9). These mismatched properties may hinder coordinated drug exposure at the infection site and limit the therapeutic potential of the combination.

Drug modification and formulation strategies based on covalent conjugation or non-covalent complexation have been widely used to improve the physicochemical and pharmacokinetic properties of therapeutic agents (10, 11). Among these strategies, covalent conjugation is particularly relevant to COL because its multiple amino groups provide reactive sites for coupling with carboxyl-containing functional molecules. For example, a COL-hyaluronic acid (HA) conjugate can be synthesized via carbodiimide chemistry by coupling the amino groups of COL with the carboxyl groups of HA. Studies have demonstrated that the COL -hyaluronic acid (HA) conjugate exhibits significant antibacterial activity. Meanwhile, this conjugate reduced the nephrotoxicity of COL by 20%–50%, indicating that the novel COL-HA conjugate holds promising application prospects in the development of safe and effective antimicrobial nanomedicines (12). Ferguson et al. (13) found that the dextrin-COL conjugates (dextrin molecular weight: 7,500–48,000 g/mol), particularly the one with low molecular weight (~7,500 g/mol) dextrin and ~1 mol% succinoylation, exhibit antimicrobial activity comparable to colistimethate sodium against gram-negative pathogens and significantly reduce *in vitro* kidney cell toxicity, highlighting their potential for the safe treatment of MDR infections. These findings suggest that COL conjugation can be a feasible strategy to modulate drug exposure, improve safety, and preserve antibacterial efficacy.

Therefore, we performed O-carboxymethylation of the phenolic hydroxyl group of NIC to generate Axilisuan (AXLS), a carboxyl-bearing NIC derivative intended as a precursor for subsequent amide coupling with the amino groups of COL. This study further examined whether O-carboxymethylation of the phenolic hydroxyl group of NIC could improve its aqueous solubility and cytotoxicity profile while preserving its COL-potentiating activity against COL-R gram-negative bacteria. The results provide a chemical basis for the future development of COL-NIC conjugates and support phenolic hydroxyl O-carboxymethylation as a new strategy for optimizing salicylanilide-based antibacterial adjuvants.

## RESULTS AND DISCUSSION

### Synthesis of AXLS

**Fig 1:**
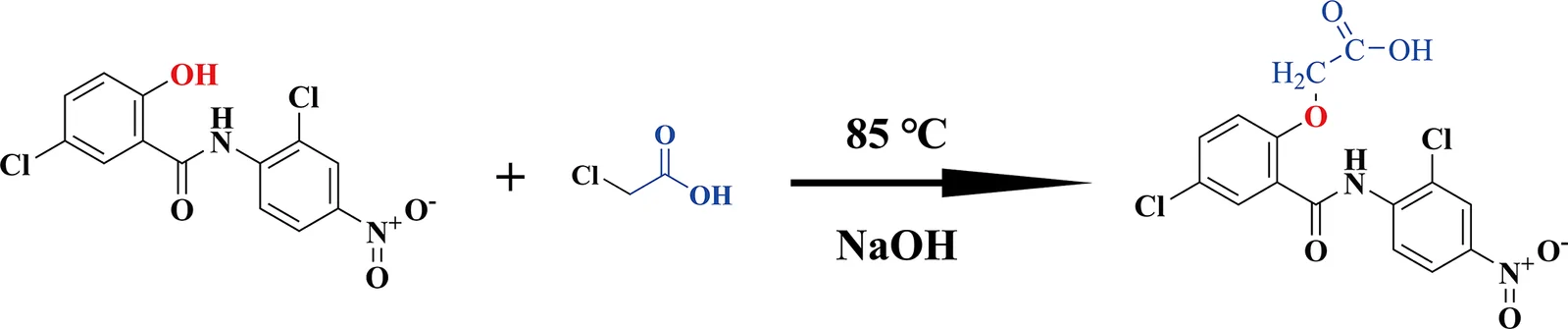
Synthesis strategy of NIC derivatization (AXLS).

Poor aqueous solubility remains a major obstacle to the use of NIC as a COL-potentiating adjuvant (14, 15). Lu et al. (16) previously reported that modification of the phenolic hydroxyl group of NIC, including O-glycosylation/galactosylation and O-amino acid esterification, did not abolish its synergistic interaction with COL. Unlike previously reported phenolic hydroxyl substitutions, we introduced a carboxyl-containing side chain onto the phenolic oxygen of NIC through O-carboxymethylation to obtain AXLS (Fig. 1). The introduction of an ionizable carboxyl-containing side chain is a well-established medicinal chemistry strategy for increasing molecular polarity and promoting pH-dependent aqueous solubility, while also serving as a reactive handle for subsequent amide bond formation with the amino groups of COL.

**Fig 2:**
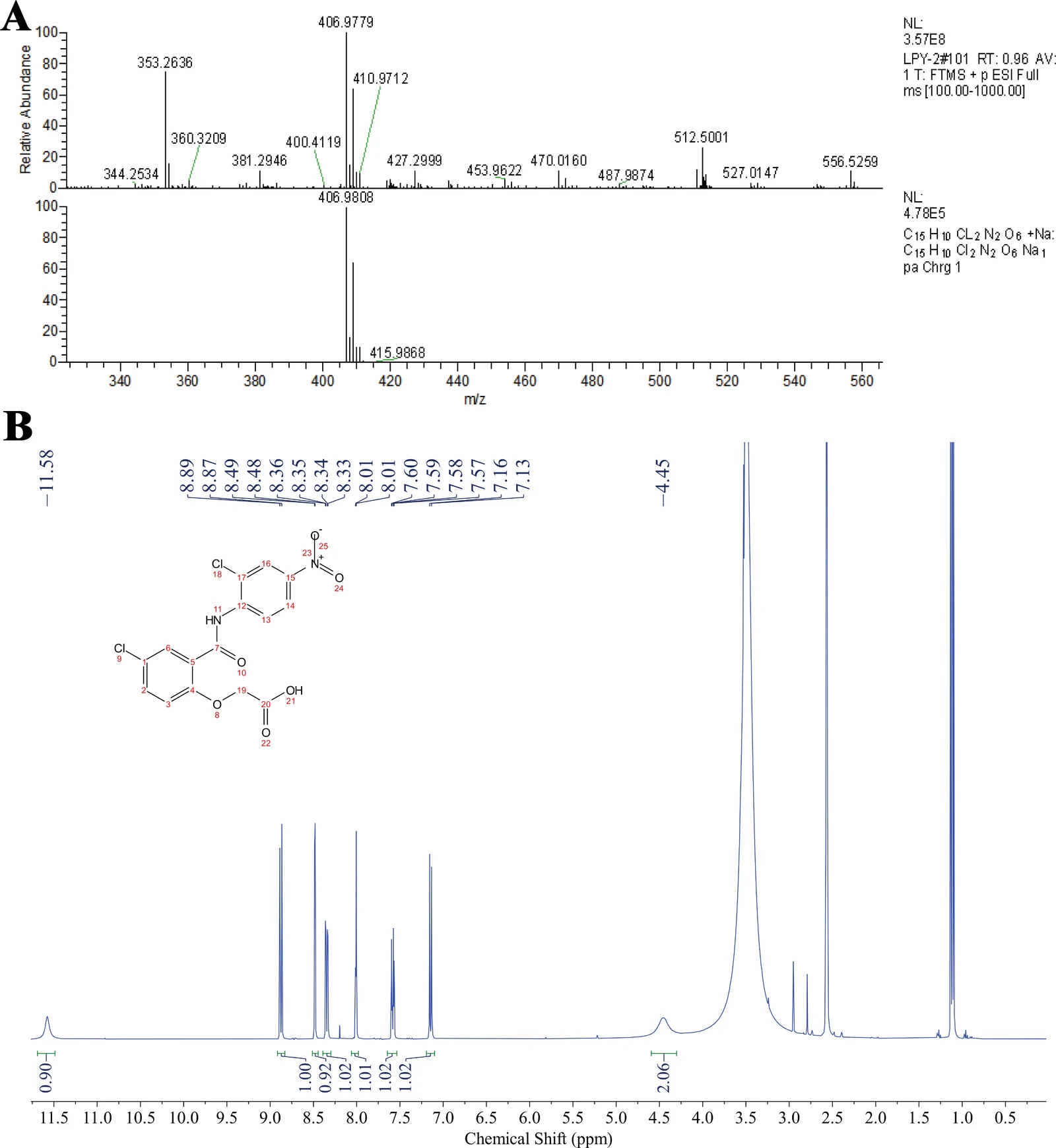
Structural characterization of AXLS. (**A**) HRMS of AXLS. (**B**) ^1^H NMR (400 MHz, DMSO-*d*_6_) spectrum of AXLS.

The structure of AXLS [5-chloro-2-(carboxymethoxy)-N-(2-chloro-4-nitrophenyl)benzamide] was confirmed by proton nuclear magnetic resonance (^1^H NMR) and high-resolution mass spectrometry (HRMS) (Fig. 2). The ^1^H NMR spectrum of AXLS in DMSO-*d*₆ showed characteristic aromatic proton signals at δ 8.88 (d, *J* = 9.2 Hz, 1H), 8.48 (d, *J* = 2.6 Hz, 1H), 8.35 (dd, *J* = 9.3, 2.6 Hz, 1H), 8.01 (d, *J* = 2.8 Hz, 1H), 7.58 (dd, *J* = 8.8, 2.8 Hz, 1H), and 7.15 (d, *J* = 8.8 Hz, 1H). A singlet at δ 4.46 ppm was assigned to the methylene protons of the introduced carboxymethyl group, and the downfield singlet at δ 11.58 corresponded to the amide proton. HRMS showed an ion peak at *m*/*z* 406.9779, consistent with the calculated value for C_15_H_10_C_l2_N_2_O_6_Na [M + Na]^+^. These data confirmed the successful synthesis of AXLS, which was obtained in an isolated yield of 27.5%.

High-performance liquid chromatography (HPLC) analysis showed distinct retention times for NIC and AXLS at 3.143 and 6.389 min, respectively. The HPLC purity of AXLS was 98.82% based on peak-area normalization (Fig. S1). Together with the ^1^H NMR and HRMS results, these data supported the successful preparation and high purity of AXLS used in the subsequent biological experiments.

### Antibacterial activity of AXLS in combination with COL against COL-R strains *in vitro*

We first measured the antibacterial effect of AXLS alone on COL-R strains (*Salmonella*, *Escherichia coli*, *P. aeruginosa*, and *Klebsiella pneumoniae*). The minimum inhibitory concentration (MIC) values of AXLS for all tested strains were ≥256 mg/L, indicating that AXLS alone exhibited no antibacterial activity against the COL-R isolates. We performed checkerboard assays to determine whether AXLS enhanced the antibacterial activity of COL against the tested strains. The fractional inhibitory concentration index (FICI) values for all strains are summarized in Table 1. The results showed that the combination exhibited synergistic activity against all COL-R strains (FICI < 0.5). When AXLS was combined with COL at 2 mg/L, the minimum resistance reversal concentration (MRC) of AXLS was 0.03125–0.25 mg/L (Table 2), corresponding to a 1,024- to 16,384-fold reduction relative to the MIC of AXLS alone. Compared with NIC, AXLS showed comparable MRCs in most strains, indicating that O-carboxymethylation did not impair NIC’s COL-potentiating activity. These results further demonstrated the synergistic antibacterial activity between AXLS and COL against COL-R strains and indicated that AXLS reversed COL resistance in COL-R strains at very low concentrations. Our findings indicated that O-carboxymethylation at the phenolic hydroxyl group of NIC did not affect its synergistic activity with COL against COL-R GNB. These results, consistent with previous reports, demonstrated that substitution of the hydroxyl group in NIC likewise preserved its synergistic effect with COL against COL-R GNB (16).

**TABLE 1:** Minimum inhibitory concentration (MIC) and fractional inhibitory concentration index (FICI) values of AXLS and COL

Strain	*mcr-1*	MIC (mg/L) alone		MICs in combination (COL/AXLS, mg/L)	FICI
		COL	AXLS		
*E. coli*					
3E	+	8	>512	0.5/4	<0.07031
Y150	+	16	>512	0.5/32	<0.09375
16E	+	8	>512	0.25/4	<0.03906
L190	−	8	>512	0.25/2	<0.03515
L157	−	8	>512	0.25/4	<0.03906
L58	−	4	>512	0.25/4	<0.07031
*Salmonella*					
SH30	+	8	>512	0.5/16	<0.09375
F30	+	16	>512	0.5/4	<0.03906
SH05	+	16	>512	0.5/2	<0.03516
290	+	8	>512	0.5/8	<0.07813
11R	−	16	>512	0.5/2	<0.03516
MLG	−	8	>512	0.25/4	<0.03906
*K. pneumoniae*					
3Q	+	8	>512	0.5/16	<0.09375
3F	+	8	>512	0.5/16	<0.09375
5G	+	8	>512	0.25/2	<0.03515
1F	+	16	>512	0.5/4	<0.03906
1Q	+	8	>512	0.5/32	<0.125
KP9	−	8	>512	0.25/4	<0.03906
*A. baumannii* 18D	−	8	>512	0.5/32	<0.125
*P. aeruginosa*					
H4	+	8	256	0.5/4	0.07813
H091	+	8	256	0.25/4	0.04688
H8	+	8	256	0.5/4	0.07813
H081	−	8	256	0.25/1	0.03515

**TABLE 2:** Minimum resistance reversal concentration (MRC) of AXLS and NIC

Strain	*mcr-1*	MRC (mg/L)	
		AXLS	NIC
*E. coli*			
3E	+	0.0625	0.0625
Y150	+	0.03125	0.0625
16E	+	0.03125	0.01562
L190	–	0.03125	0.03125
L157	–	0.125	0.0625
L58	–	0.0625	0.0625
*Salmonella*			
SH30	+	0.03125	0.0625
F30	+	0.03,125	0.0625
SH05	+	0.03125	0.03125
290	+	0.0625	0.125
11R	–	0.03125	0.03125
MLG	–	0.03125	0.0625
*K. pneumoniae*			
3Q	+	0.03125	0.0625
3F	+	0.0625	0.0625
5G	+	0.125	0.5
1F	+	0.03125	0.125
1Q	+	0.03125	0.125
KP9	–	0.03125	0.0625
*A. baumannii* 18D	–	0.125	0.03125
*P. aeruginosa*			
H4	+	0.25	0.5
H091	+	0.25	0.5
H8	+	0.25	0.5
H081	–	0.25	0.5

### Time-killing curves of different concentrations of AXLS combined with COL against COL-R strains

**Fig 3:**
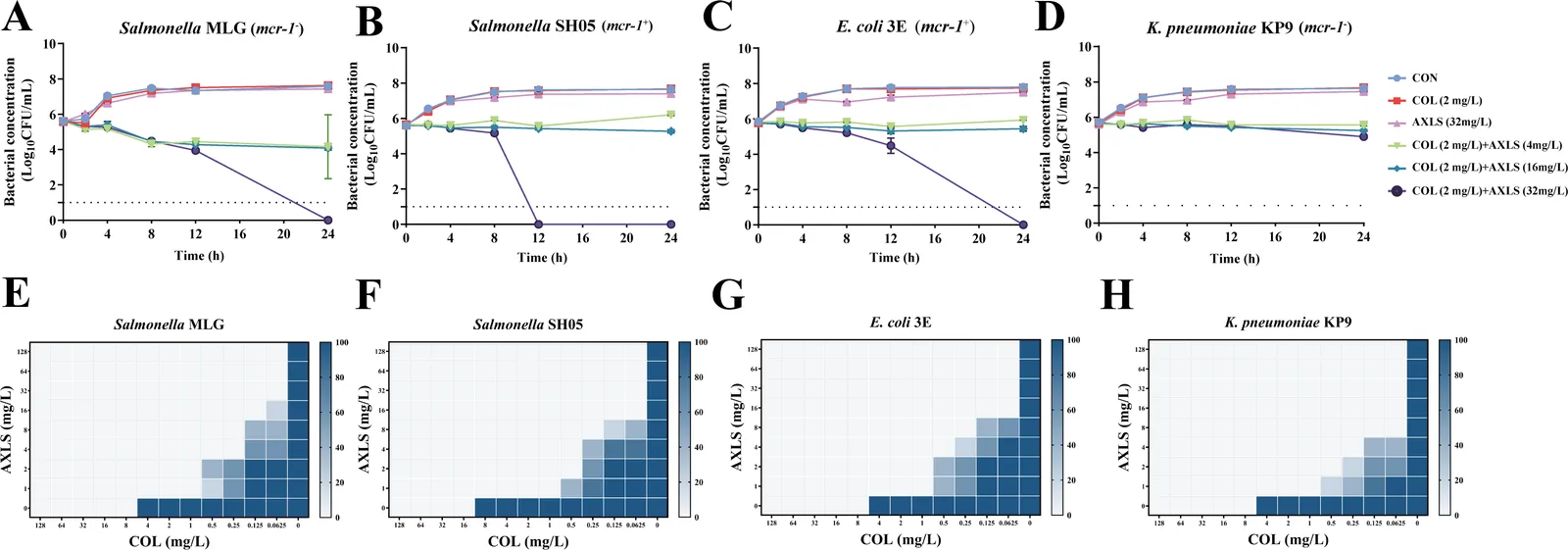
Synergistic antibacterial activity of AXLS in combination with colistin against colistin-resistant Enterobacteriaceae. (**A–D**) Time-kill curves showing antibacterial activity of AXLS in combination with COL against *E. coli* 3E, *Salmonella* SH05, and *Salmonella* MLG, *K. pneumoniae* KP9, respectively. The horizontal dashed line represents the limit of detection (1 log_10_ CFU/mL). Synergy was defined as a ≥2 log_10_ CFU/mL reduction with the combination compared with the most active single agent at 24 h. Data are presented as the mean ± SD from three independent biological experiments. (**E–H**) Representative heat plots of the microdilution checkerboard assay for the combination of COL and AXLS against COL-R strains.

To further evaluate the synergistic activity of AXLS and COL, time-kill assays were performed using *E. coli* 3E (*mcr-1*^+^), *Salmonella* SH05 (*mcr-1*^+^), *Salmonella* MLG (*mcr-1*^–^), *K. pneumoniae* KP9 (*mcr-1*^–^), *P. aeruginosa* H081 (*mcr-1*^–^), and *P. aeruginosa* H8 (*mcr-1*^+^) (Fig. 3 and Fig. S2). Compared with the blank control, monotherapy neither caused a substantial reduction in viable bacterial counts nor exerted bactericidal effects against the tested strains. In contrast, at 24 h, the combination of COL (2 mg/L) and AXLS (4, 16, or 32 mg/L) reduced viable counts by ≥2 log_10_ CFU/mL compared with the most active single agent across all tested strains. This indicated synergy between AXLS and COL.

### Solubility studies

**Fig 4:**
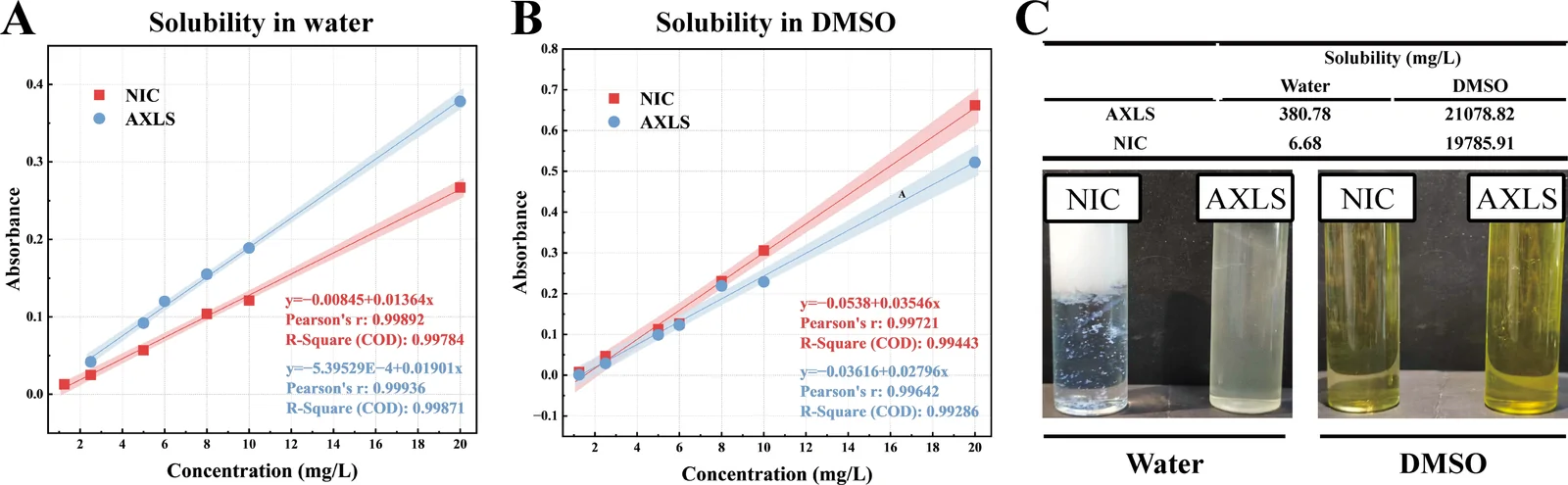
Solubility test. (**A**) The solubility curves of AXLS and NIC in water. (**B**) Solubility curves of AXLS and NIC in DMSO. (**C**) The solubility of excessive AXLS and NIC in water (DMSO).

In addition, UV spectrophotometry was employed to investigate the solubility of AXLS in unbuffered deionized water (pH 6.5) and dimethyl sulfoxide (DMSO). First, the characteristic ultraviolet absorption wavelengths of NIC and AXLS were determined. Subsequently, the standard curves were constructed in water and DMSO, respectively (Fig. 4). The calibration curves showed good linearity between absorbance and concentration within the detected concentration range. Ultraviolet spectrophotometric analysis revealed that AXLS exhibited substantially improved aqueous solubility compared with NIC. The solubilities of NIC and AXLS in water were 6.68 and 380.78 mg/L, respectively, indicating an approximately 57-fold increase for AXLS (*P* < 0.001). By contrast, their solubilities in DMSO were comparable, with values of 19,785.91 and 21,078.82 mg/L, respectively (*P* > 0.05) (Fig. 4).

### AXLS shows lower hemolytic and cytotoxicity than NIC

**Fig 5:**
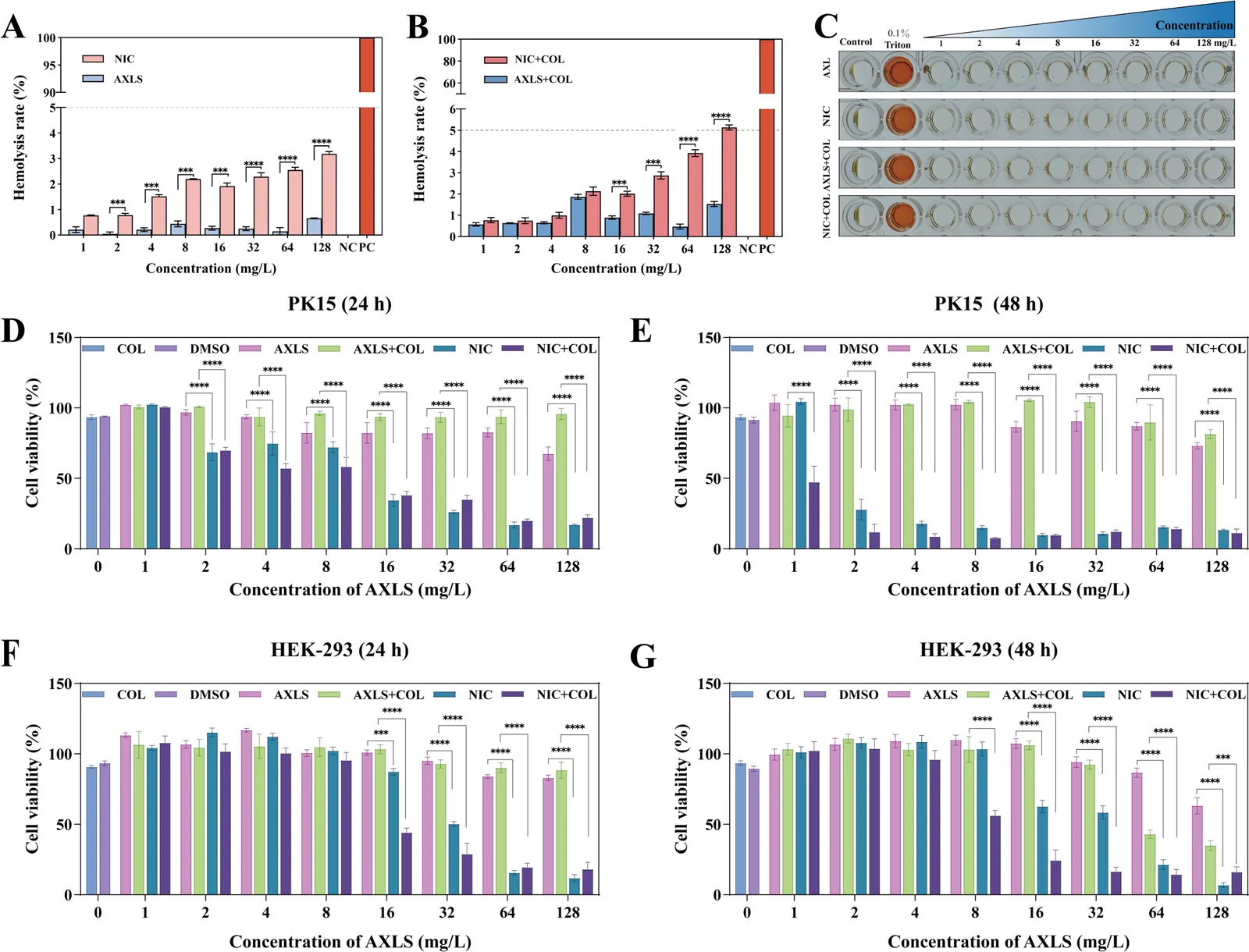
Safety assessment. (**A**) Hemolytic effects of AXLS and NIC on mouse erythrocytes. (**B**) Hemolytic effects of AXLS or NIC in combination with COL on mouse erythrocytes. (**C**) Representative images of erythrocyte suspensions following treatment with AXLS, NIC, AXLS + COL, or NIC + COL at the indicated concentrations. (**D and E**) Cell viability of PK-15 cells following treatment with COL, AXLS, AXLS + COL, NIC, or NIC + COL for 24 and 48 h, respectively. (**F and G**) Cell viability of HEK-293 cells under the same treatment conditions for 24 and 48 h, respectively. Data are presented as the mean ± SD of three independent biological experiments (*n* = 3), each performed in technical triplicate. Statistical analysis was performed using two-way ANOVA followed by Tukey’s multiple comparisons test. At each tested concentration, AXLS was compared with NIC, and AXLS in combination with COL was compared with NIC in combination with COL. ***P < 0.001 and ****P < 0.0001.

Safety assessment is a critical component for evaluating the clinical application potential of drugs (17). To further evaluate the application potential of AXLS as a single agent and in combination with COL, systematic safety assessments were conducted using mouse red blood cells (RBCs), mammalian kidney cells (PK-15 cell line), and human-derived kidney cells (HEK-293 cell line). The results showed that neither AXLS nor NIC alone induced hemolysis of mouse RBCs at concentrations ranging from 1 to 128 mg/L. Furthermore, at matched concentrations, AXLS alone and the combination of AXLS and COL showed significantly lower cytotoxicity toward PK-15 cells than the corresponding NIC-alone and NIC-COL treatments, respectively (Fig. 5D and E). Moreover, cytotoxicity assays on the HEK-293 cell line demonstrated that both AXLS monotherapy and AXLS combination with COL, at higher concentrations, exhibited higher cell viability compared to the corresponding NIC treatment groups (Fig. 5F and G). Notably, co-administration of 2 mg/L COL with AXLS at various concentrations did not cause hemolysis of mouse RBCs, whereas the combination of 2 mg/L COL with NIC at concentrations 128 mg/L resulted in evident RBC hemolysis. These findings indicated that AXLS not only exhibited favorable hemocompatibility and cellular safety as a single agent at 1–128 mg/L but also demonstrated superior safety compared with NIC when combined with COL.

### Mechanisms of colistin potentiation by AXLS

**Fig 6:**
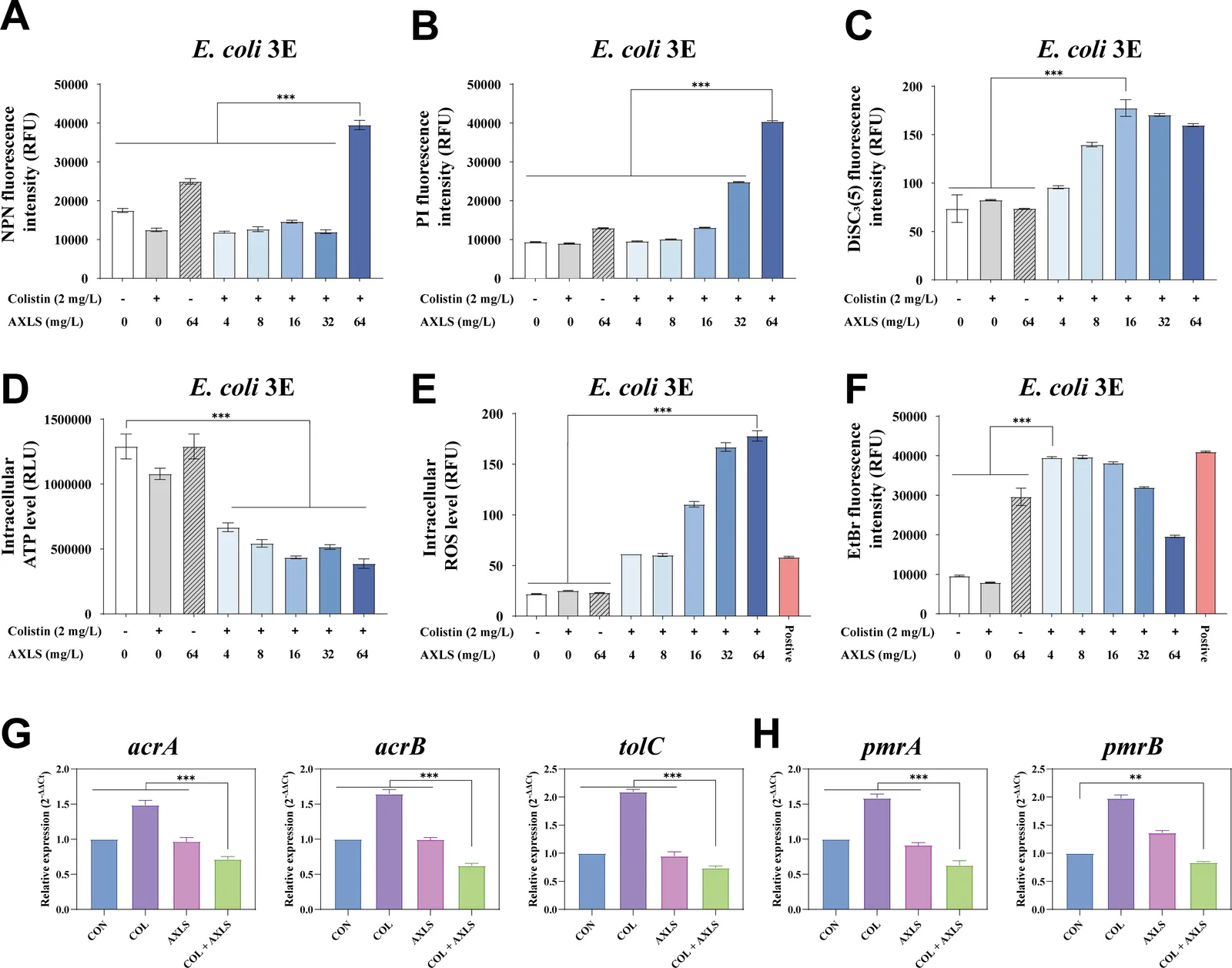
Effects of AXLS and COL on bacterial membrane function, cellular homeostasis, efflux activity, and resistance-associated gene expression in *E. coli* 3E. (**A**) Outer membrane permeability was assessed by NPN uptake. (**B**) Inner membrane integrity was assessed by PI fluorescence. (**C**) DiSC_3_(5) fluorescence was measured to assess changes in membrane potential. (**D**) Intracellular ATP levels were measured using an ATP assay. (**E**) Intracellular ROS levels were determined using DCFH-DA. (**F**) EtBr accumulation was measured to assess bacterial efflux activity, with CCCP used as a positive control. (**G**) Relative transcript levels of *acrA*, *acrB*, and *tolC* in *E. coli* 3E were determined by RT-qPCR. (**H**) Relative transcript levels of *pmrA* and *pmrB* in *E. coli* 3E were determined by RT-qPCR. Data are presented as the mean ± SD of three independent biological experiments (*n* = 3). Statistical significance was determined using one-way ANOVA followed by Tukey’s multiple comparisons test. **P < 0.01, ***P < 0.001.

The outer membrane (OM) of gram-negative bacteria acts as an effective permeability barrier against many antimicrobial agents. COL exerts its antibacterial activity through electrostatic interactions with the negatively charged phosphate groups of the lipid A moiety of lipopolysaccharide, thereby destabilizing the OM and increasing membrane permeability (18). In COL-R bacteria, lipid A modification can reduce COL binding and weaken its membrane-disrupting activity (19). We hypothesized that AXLS might restore the ability of COL to disrupt the bacterial membrane. To test this hypothesis, we assessed OM permeability using the 1-N-phenylnaphthylamine (NPN) uptake assay in *Salmonella* SH05 and *E. coli* 3E following treatment with AXLS alone, COL alone, or their combination. Compared with either agent alone, the combined treatment markedly increased NPN fluorescence in both strains, particularly at higher AXLS concentrations (Fig. 6A and Fig. S3A). These results showed that AXLS enhanced the ability of COL to permeabilize the OM in the tested strains.

The inner membrane (IM) is a selectively permeable barrier that is essential for cellular homeostasis (20). We therefore measured propidium iodide (PI) uptake to determine whether the increase in OM permeability was accompanied by loss of IM integrity. COL or AXLS alone produced only limited changes in PI fluorescence. In contrast, the combination of AXLS and COL increased PI fluorescence, particularly at AXLS concentrations of 32–64 mg/L (Fig. 6B and Fig. S3B). NIC combined with COL was previously shown to increase OM permeability (21). In the present study, the combination of AXLS and COL compromised both OM and IM integrity, indicating that damage to both membranes contributed to the potentiation of COL by AXLS in the tested strains.

To determine whether the observed membrane damage was accompanied by changes in bacterial membrane potential, we performed a DiSC_3_(5) assay. DiSC_3_(5) fluorescence was markedly higher in bacteria treated with the combination of AXLS and COL than in untreated bacteria or those treated with either agent alone (Fig. 6C and Fig. S3C). This increase indicated dissipation of the membrane potential (Δψ) (22). Because Δψ is a major component of the proton motive force, its dissipation can impair ATP synthesis and thereby affect many ATP-dependent cellular processes (22). We therefore measured intracellular ATP levels to determine whether the combination of AXLS and COL also disrupted cellular energy homeostasis. Intracellular ATP levels were significantly lower after treatment with the combination of AXLS and COL than after treatment with AXLS alone or COL alone (Fig. 6D and Fig. S3D). At a fixed COL concentration, ATP levels decreased further as the AXLS concentration increased. These findings indicated that the combination of AXLS and COL impaired cellular energy homeostasis at the concentrations tested. Because membrane depolarization and impaired energy metabolism are often associated with oxidative stress, we next measured intracellular reactive oxygen species (ROS) levels (23). ROS levels were markedly higher after exposure to both agents than after exposure to either agent alone (Fig. 6E and Fig. S3E). Excessive ROS can damage membrane lipids, proteins, and DNA, thereby disrupting cellular homeostasis and contributing to bacterial death (23). Similar membrane depolarization, ATP depletion, and ROS accumulation were previously reported when NIC was combined with COL (21). These similarities indicated that AXLS retained these effects of NIC after O-carboxymethylation.

Efflux pumps are important determinants of intracellular drug accumulation. We next assessed bacterial efflux activity using an ethidium bromide (EtBr) accumulation assay, with the known efflux pump inhibitor CCCP included as a positive control (24). At AXLS concentrations of 8–32 mg/L, the combination of AXLS and COL resulted in substantially higher EtBr fluorescence than treatment with AXLS alone or COL alone in both *Salmonella* SH05 and *E. coli* 3E (Fig. 6F and Fig. S3F). The increased intracellular accumulation of EtBr showed that the combination of AXLS and COL inhibited bacterial efflux activity in *Salmonella* SH05 and *E. coli* 3E under the conditions tested. A similar increase in EtBr accumulation was previously reported for the combination of NIC and COL (21). The increase in EtBr accumulation observed with the combination of AXLS and COL therefore suggested that the efflux-inhibitory effect of NIC was retained after O-carboxymethylation.

AcrAB-TolC is a major RND-family multidrug efflux system in gram-negative bacteria. In this system, AcrB functions as the inner membrane transporter, AcrA as the periplasmic adaptor protein, and TolC as the outer membrane channel (25). To further investigate the mechanism underlying the inhibition of efflux activity, we measured the transcript levels of *acrA*, *acrB*, and *tolC* in *E. coli* 3E by reverse transcription quantitative PCR (RT-qPCR). The combination of AXLS and COL significantly reduced the transcript levels of all three genes compared with the untreated control (Fig. 6G). Together with the increased intracellular accumulation of EtBr, these results indicated that the combination of AXLS and COL inhibited the expression and function of the AcrAB-TolC efflux system in *E. coli* 3E.

PmrAB is a key two-component regulatory system that contributes to COL resistance by regulating lipid A modification (26). RT-qPCR showed that the combination of AXLS and COL significantly reduced the transcript levels of both *pmrA* and *pmrB* (Fig. 6H). These results indicated that the combination of AXLS and COL suppressed PmrAB signaling associated with COL resistance in *E. coli* 3E. This suppression may reduce lipid A modification and thereby enhance the ability of COL to disrupt the bacterial membrane. Changes in the PmrAB system were not reported in the previous study of NIC combined with COL (21). The present findings indicated that suppression of the PmrAB pathway is an additional mechanism by which AXLS potentiates COL in *E. coli* 3E.

Together, these findings showed that AXLS retained several colistin-potentiating effects of NIC after O-carboxymethylation. When combined with COL, AXLS increased membrane permeability, dissipated the membrane potential, reduced intracellular ATP levels, increased ROS accumulation, and inhibited bacterial efflux activity. Similar effects were previously reported for NIC combined with COL (21). However, unlike NIC, AXLS also disrupted IM integrity when combined with COL. In *E. coli* 3E, the combination of AXLS and COL also reduced the transcription of genes involved in AcrAB-TolC-mediated efflux and PmrAB-associated COL resistance. Collectively, these results provided a mechanistic basis for the potentiation of COL by AXLS in the tested strains. Further studies are needed to determine whether these effects also occur at AXLS concentrations close to the MRC.

### Limitations

This study has two main limitations. All antibacterial and mechanistic experiments were conducted *in vitro*, without evaluation of *in vivo* efficacy or pharmacokinetics. It remains unclear whether effective AXLS concentrations can be achieved *in vivo*. Further pharmacokinetic and infection-model studies are needed to evaluate combined treatment with AXLS and COL *in vivo*.

In addition, the mechanistic experiments were conducted using high concentrations of AXLS. Therefore, the mechanisms underlying COL potentiation at AXLS concentrations close to the MRC require further investigation.

### Conclusions

In conclusion, we synthesized AXLS, a novel O-carboxymethyl derivative of NIC that retained synergistic activity with COL against COL-R GNB. Compared with NIC, AXLS showed lower cytotoxicity and greater aqueous solubility. Mechanistic analyzes in *E. coli* 3E and *Salmonella* SH05 showed that the combination of AXLS and COL enhanced membrane damage, dissipated the membrane potential, disrupted cellular energy homeostasis, increased ROS accumulation, and inhibited bacterial efflux. In *E. coli* 3E, these effects were also accompanied by downregulation of the AcrAB-TolC efflux system and PmrAB-associated COL resistance pathway. Overall, this work provides critical insights for the future development and optimization of NIC-based strategies to overcome COL resistance, provides a reference for the chemical modification of salicylanilide derivatives as antibacterial adjuvants, and establishes a rational foundation for the development of novel COL-NIC conjugates targeting COL-R gram-negative bacterial infections. Nevertheless, all assessments in this study were performed exclusively *in vitro*, and mechanistic assays were carried out at high AXLS concentrations. Future work should establish whether these effects persist at AXLS concentrations near the MRC, characterize the pharmacokinetic profiles of AXLS when used together with COL, and evaluate the therapeutic efficacy of this combination in animal infection models.

## MATERIALS AND METHODS

### Bacterial strains and culture conditions

This study selected 23 COL-R strains, comprising both *mcr-1*-negative and *mcr-1*-positive isolates, including 6 strains of *Salmonella*, 6 strains of *Escherichia coli*, 6 strains of *Klebsiella pneumoniae*, 4 strains of *Pseudomonas aeruginosa*, and 1 strain of *Acinetobacter baumannii*. All strain identifications were confirmed by PCR analysis and 16S rRNA sequencing, along with matrix-assisted laser desorption/ionization time-of-flight mass spectrometry detection (AXIMA Performance; Shimadzu Corporation, Kyoto, Japan). COL susceptibility was interpreted according to the European Committee on Antimicrobial Susceptibility Testing clinical breakpoint criteria (version 11.0, 2021), with MICs > 2 mg/L classified as resistant (27). COL was procured from China Shengxue Dacheng Pharmaceutical. COL was dissolved in water, while AXLS was dissolved in a 1:1 (vol/vol) DMSO-water mixture. Both compounds were prepared as stock solutions at 5,120 mg/L, respectively, each yielding a final concentration of 5,120 mg/L. All chemical reagents and solvents used were of analytical grade.

### Synthesis of AXLS

AXLS was synthesized from NIC via an alkylation reaction, as illustrated in Fig. 1. Commercial niclosamide was dissolved in an aqueous sodium hydroxide solution under stirring until complete dissolution. Subsequently, an appropriate amount of sodium chloroacetate was added, and the reaction mixture was heated to 85°C. The reaction progress was monitored by thin-layer chromatography until completion. The pH of the reaction solution was then adjusted with hydrochloric acid until full precipitation occurred, and the precipitate was collected by filtration to afford the crude product, in which the phenolic hydroxyl group of NIC was substituted by a carboxymethyl moiety. The crude product was recrystallized from 75% ethanol to yield the target compound AXLS.

### Analytical methods

High-resolution mass spectrometry (HRMS) of AXLS was performed using a Dionex Ultimate 3000 system coupled with a Q-Exactive Orbitrap mass spectrometer (Thermo Fisher Scientific, Bremen, Germany). ^1^H NMR spectra were recorded on a Bruker DPX-400 spectrometer in DMSO-d₆, using tetramethylsilane as an internal standard.

HPLC analysis was performed using an Agilent 1260 Infinity II HPLC system equipped with a C18 column. Isocratic elution was performed with methanol/0.1% formic acid in water (70:30, vol/vol) at a flow rate of 1.0 mL/min. The column temperature was 30 °C, the injection volume was 10 μL, and detection was performed at 330 nm. Samples were dissolved in absolute ethanol. The total run time was 10 min. Chromatographic purity was calculated by peak-area normalization.

### Susceptibility testing

The minimum inhibitory concentrations (MICs) of AXLS and COL were determined using the broth microdilution method according to CLSI M07 guidelines (28). COL and AXLS were serially diluted in Mueller-Hinton broth (MHB) and subjected to serial dilution in 96-well plates (29, 30). Bacterial suspensions were adjusted to the 0.5 McFarland standard and subsequently diluted 1:200 in culture medium. Following overnight static incubation at 37°C, the MIC was recorded as the lowest antibiotic concentration that prevented visible bacterial growth. MIC assays were performed in three independent experiments, with three technical replicates per experiment.

### The minimum resistance reversal concentration

The MRC of AXLS, defined as the lowest concentration of AXLS that inhibited bacterial growth at a COL concentration of 2 mg/L, was determined using the broth microdilution method (31). The samples were incubated statically at 37°C in a constant-temperature biochemical incubator for 18 h. The MIC was then determined according to standard criteria, and the experiment was repeated in triplicate for parallel validation.

### Checkerboard assays

To determine the interaction between AXLS and COL, checkerboard assays were performed. Twofold serial dilutions of COL were prepared along the *x*-axis, whereas twofold serial dilutions of AXLS were prepared along the *y*-axis to generate a concentration matrix. Overnight bacterial cultures were diluted in saline to an OD_600_ of 0.5 and then further diluted in MHB. The diluted bacterial suspension was added to each well to achieve a final inoculum of approximately 5 × 10^5^ CFU/mL. Wells containing MHB with or without bacterial cells were included as growth and sterility controls, respectively. The final volume in each well was 70 μL. Plates were incubated statically at 37°C for 18 h before fractional inhibitory concentration index (FICI) determination. Checkerboard assays were performed in three independent biological experiments. The fractional inhibitory concentration index (FICI) was calculated according to the following formula:


$$FICI=\frac{MIC_{A+B}}{MIC_{A}}+\frac{MIC_{B+A}}{MIC_{B}}$$

where MIC_A_ is the MIC of A alone, MIC_B_ is the MIC of B alone, MIC_A+B_ is the MIC of A in combination with B, and MIC_B+A_ is the MIC of B in combination with A.

The interaction was classified as synergistic when FICI ≤ 0.5, additive when 0.5 < FICI ≤ 1, indifferent when 1 < FICI ≤ 2, and antagonistic when FICI > 2.

### Time-kill curve analysis

Time-kill assays were performed on *E. coli* 3E (*mcr-1*^+^), *Salmonella* SH05 (*mcr-1*^+^), *Salmonella* MLG (*mcr-1*^−^), *K. pneumoniae* KP9 (*mcr-1*^−^), *P. aeruginosa* H8 (*mcr-1*^+^), and *P. aeruginosa* H081 (*mcr-1*^−^) in Luria-Bertani (LB) broth. The treatment groups included COL (2 mg/L) alone, AXLS (32 mg/L) alone, and the combination of AXLS (4, 16, or 32 mg/L) and COL (2 mg/L). Overnight bacterial cultures were diluted 1:100 in fresh LB broth to a final concentration of 1 × 10⁵ CFU/mL. Then, the samples and a drug-free control group were incubated with shaking at 37°C and 150 rpm for 24 h. Aliquots (100 µL) of the bacterial suspensions were collected at predetermined intervals following inoculation (0, 2, 4, 8, 12, and 24 h), serially diluted in phosphate-buffered saline, and plated onto LB agar for viable count determination after incubation at 37°C for 12 h, with results expressed as log_10_ CFU/mL. Synergy in the time-kill assay was defined as a ≥2 log_10_ CFU/mL reduction in viable bacterial counts for the combination treatment compared with the most active single agent at 24 h (32, 33).

### Safety assessment

Whole blood samples were collected in a centrifuge tube containing an anticoagulant agent (heparin sodium). Mouse erythrocyte suspensions were treated with various concentrations (1–128 mg/L) of AXLS or NIC at 37°C for 1.5 h. For combination treatment, erythrocyte suspensions were co-incubated with COL (2 mg/L) together with AXLS or NIC (1–128 mg/L) under the same conditions. Subsequently, the absorbance of the supernatant was measured at 540 nm, and the hemolysis rate of each sample was calculated through comparison with the positive control. Cell viability was assessed using the Enhanced Cell Counting Kit-8 (CCK-8, Cat. No. BA00208, Biossusa, China) (34–38). Logarithmic phase cells (100 μL, 1 × 10⁵ cells/well) were seeded into 96-well plates, with normally cultured cells as the negative control. A vehicle control containing 1.25% DMSO, corresponding to the final DMSO concentration in the 128 mg/L AXLS and NIC treatment groups, was included to control for the effect of DMSO on cell viability. After 24 and 48 h of culture, 10 μL of CCK-8 reagent was added to each well, followed by another 2 h of incubation.

### Solubility test

The solubilities of AXLS and NIC were determined by ultraviolet spectrophotometry (UV spectrophotometry) following an equilibrium solubility method. An excess of AXLS or NIC was added separately to unbuffered deionized water (pH 6.5) or DMSO. The resulting samples were stirred continuously at 37°C for 24 h to reach equilibrium. After incubation, the suspensions were centrifuged at high speed to remove undissolved solids. The supernatants were subsequently filtered through a 0.22 μm membrane filter. The filtrates were appropriately diluted when necessary, and the absorbance was measured at 330 nm using the corresponding solvent as a blank. Calibration curves for AXLS and NIC were constructed in advance using standard solutions with known concentrations, and good linearity was confirmed (*R*² > 0.99). The solubility of each compound was calculated based on the corresponding calibration curve. All measurements were performed in triplicate.

### Fluorescent probe assays

The effects of AXLS on COL-mediated membrane perturbation and intracellular dye accumulation were evaluated using fluorescence-based assays with *Salmonella* SH05 and *E. coli* 3E. Briefly, overnight cultures were diluted in fresh LB broth and incubated at 37°C with shaking until the mid-logarithmic phase was reached. Bacterial cells were collected by centrifugation, washed twice with 1 mM HEPES buffer (pH 7.0), and resuspended in the same buffer to an OD_600_ of approximately 0.5. The bacterial suspensions were exposed to COL at 2 mg/L, AXLS alone, or COL combined with AXLS at the indicated concentrations. Untreated bacteria were used as the negative control, and positive controls were included where appropriate.

Membrane permeability was evaluated using 1-N-phenylnaphthylamine (NPN) and propidium iodide (PI) to assess outer membrane and IM integrity, respectively. Bacterial suspensions adjusted to OD_600_ ≈ 0.5 were incubated with NPN or PI in the dark at 37°C, followed by treatment with COL, AXLS, or their combinations. NPN was used at a final concentration of 10 μM, and fluorescence was measured at excitation/emission wavelengths of 350/420 nm. For the PI assay, fluorescence intensity was recorded at excitation/emission wavelengths of 535/615 nm after incubation. Increased NPN and PI fluorescence indicates enhanced outer membrane permeabilization and loss of IM integrity, respectively.

Ethidium bromide (EtBr) accumulation was measured to evaluate intracellular dye retention. Bacterial suspensions were incubated with EtBr at a final concentration of 5 μM and treated with COL, AXLS, or their combinations. Carbonyl cyanide m-chlorophenyl hydrazone (CCCP) was used as a positive control when applicable. Fluorescence was detected at an excitation wavelength of 530 nm and an emission wavelength of 600 nm. Increased EtBr fluorescence is considered to reflect enhanced intracellular accumulation of EtBr, which may result from increased membrane permeability and/or reduced dye efflux.

Membrane potential in *Salmonella* SH05 and *E. coli* 3E was measured using 0.5 µM 3,3′-dipropylthiadicarbocyanine iodide [DiSC_3_(5)]. Fluorescence was recorded at excitation and emission wavelengths of 622 and 670 nm, respectively.

Intracellular ATP levels were measured using an Enhanced ATP Assay Kit (Beyotime, China), and the luminescence of the supernatant was recorded using a Spark 10M microplate reader (Tecan, Switzerland).

Intracellular reactive oxygen species (ROS) levels in *Salmonella* SH05 and *E. coli* 3E were measured using 10 µM DCFH-DA (Beyotime, China). Fluorescence was recorded at excitation and emission wavelengths of 488 and 525 nm, respectively.

All fluorescence assays were performed with at least three independent biological experiments (*n* = 3), and the results are presented as the mean ± SD.

### Reverse transcription quantitative PCR

Overnight cultures of *E. coli* 3E were treated for 4 h with AXLS (4 mg/L), COL (2 mg/L), or their combination (4 mg/L AXLS and 2 mg/L COL). Total RNA was extracted using RNAiso Plus, and equal amounts of RNA were reverse-transcribed into cDNA using the PrimeScript RT Reagent Kit (TaKaRa). Quantitative PCR was performed using ChamQ Universal SYBR qPCR Master Mix (Vazyme) on a 7500 Fast Real-Time PCR System (Applied Biosystems, USA). The gene-specific primers are listed in Table S1, and 16S rRNA was used as the reference gene. Each sample was analyzed in technical duplicate, and relative transcript levels were calculated using the 2^−ΔΔCt^ method.

### Statistical analysis

Statistical analyses were performed using GraphPad Prism 9 and SPSS. All data from at least three independent biological replicates are presented as the mean ± SD. Unless otherwise noted, an unpaired two-tailed *t*-test was used for comparisons between two groups, whereas one-way analysis of variance (ANOVA) followed by Tukey’s multiple comparisons test was used for comparisons among multiple groups. Cytotoxicity data at each time point, with treatment and concentration as two independent factors, were analyzed using two-way ANOVA followed by Tukey’s multiple comparisons test. A *P*-value <0.05 was considered statistically significant (*P < 0.05, **P < 0.01, ***P < 0.001, and ****P < 0.0001).

## Data Availability

All data supporting the findings of this study are included in the article and its supplemental material. Additional raw data are available from the corresponding author upon reasonable request.

## SUPPLEMENTAL MATERIAL

Supplemental material msphere.00593-26-s0001.docx Table S1; Fig. S1 to S3.

ASM does not own the copyrights to Supplemental Material that may be linked to, or accessed through, an article. The authors have granted ASM a non-exclusive, world-wide license to publish the Supplemental Material files. Please contact the corresponding author directly for reuse.
