# Supplementary material for: The synthesis of niclosamide derivative as a novel colistin adjuvant for reversing colistin resistance and its antibacterial activity with colistin in vitro
Source: mSphere. 2026 Oct 8;11(10):e00593-26. doi: 10.1128/msphere.00593-26 (PMC13711785; doi:10.1128/msphere.00593-26)
Supplement: Supplementary file 1 [file msphere.00593-26-s0001.docx]

**Supplemental Materials**

**The synthesis of niclosamide derivative (AXLS) as a novel colistin adjuvant for reversing colistin resistance and its antibacterial activity with colistin *in vitro***

Peiyi Liu^1†^, Yingbiao Li^1†^, Fanlun Ding^1†^, Dandan He^1^, Yajun Zhai^1^, Li Yuan^1^, Xianya Wang^3^, Guoyu Yang^2*^ and Gongzheng Hu^1*^

^1^ College of Veterinary Medicine, Henan Agricultural University, Zhengzhou, Henan 450046, China

^2^ College of Science, Henan Agricultural University, Zhengzhou, Henan, 450046, China

^3^ Xinxiang Hexie Animal Pharmaceutical Co., Ltd., Xinxiang, Henan, 453000, China

^*^ Corresponding author:

^1^ Gongzheng Hu, College of Veterinary Medicine, Henan Agricultural University, Zhengzhou, Henan, 450046, China

E-mail: [yaolilab@126.com](mailto:hedandan_68@126.com),

^2^ Guoyu Yang, College of Chemistry and Materials, Henan Agricultural University, Zhengzhou, Henan, 450046, China

E-mail: [yangguoyulxy@henau.edu.cn](mailto:ffff@henau.edu.cn).

^†^These authors contributed equally to this article.

**Table S1. Primer sequences for RT-qPCR.**

| Primer Name | Oligonucleotide (5′-3′) |
| --- | --- |
| *acrA* | F: TGGCGATAAGTGGCTGGTGACA  R: CGCTTGCGGCTTGCTGGTTAT |
| *acrB* | F: AGCACGCACCACTACACCGACA  R: GCTTGGCAGACGCACGAACAGA |
| *TolC* | F: CCAGCTCTTGCTTGGCGTTGT  R: CAGACCGTGCGTTCCTCCTTCA |
| *PmrA* | F: GGTCGCCGTCAGGTATGGATGA  R: CAGGGTGTTGGTCGAGGGTTCA |
| *PmrB* | F: AAGAAGATGGCGGAGCGGTCAT  R: GCAACTGTGTAATGCGGCTGAC |
| 16sRNA | F: CCTCAGCACATTGACGTTAC  R: TTCCTCCAGATCTCTACGCA |

**Figure S1. HPLC chromatograms of NIC and AXLS.**

(A) HPLC chromatogram of NIC, showing a retention time of 3.143 min. (B) HPLC chromatogram of AXLS, showing a retention time of 6.389 min. (C) Peak integration results for AXLS. The main AXLS peak accounted for 98.817% of the total integrated peak area, corresponding to an HPLC area purity of 98.817%. Both compounds were analyzed under the same chromatographic conditions with UV detection at 330 nm.

**Figure S2. Time kill activity of AXLS in combination with COL against colistin resistant *Pseudomonas aeruginosa*.**

Time kill curves of (A) an *mcr-1*-positive *P. aeruginosa* strain H8 and (B) an *mcr-1-*negative *P. aeruginosa* strain H081. Bacterial counts were determined at the indicated time points in the untreated control and following treatment with AXLS alone, COL alone, or AXLS and COL. Synergy was defined as a ≥2 log_10_ CFU/mL reduction with the combination compared with the most active single agent at 24 h. Data are presented as the mean ± SD of three independent biological experiments (n = 3).

**Fig S3. Effects of AXLS and COL on bacterial membrane function, cellular homeostasis, and efflux activity in *Salmonella* SH05.**

(A) Outer membrane permeability was assessed by NPN uptake. (B) Inner membrane integrity was assessed by PI fluorescence. (C) DiSC_3_(5) fluorescence was measured to assess changes in membrane potential. (D) Intracellular ATP levels were measured using an ATP assay. (E) Intracellular ROS levels were determined using DCFH-DA. (F) EtBr accumulation was measured to assess bacterial efflux activity, with CCCP used as a positive control. Data are presented as the mean ± SD. Statistical significance was determined by one-way ANOVA. ****P* < 0.001.
